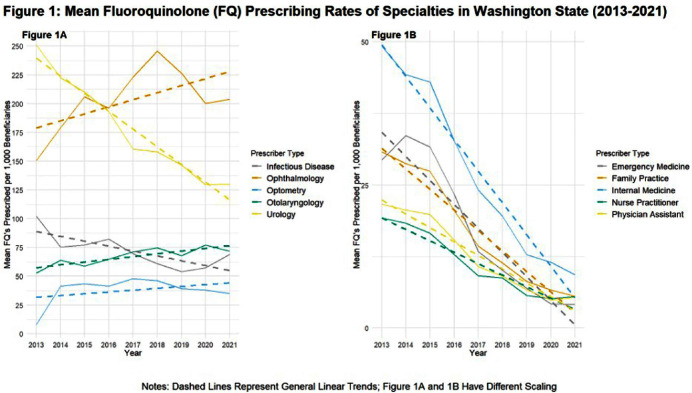# Fluoroquinolones in Focus: Unraveling Prescription Trends among Clinician Specialties in Washington State

**DOI:** 10.1017/ash.2024.144

**Published:** 2024-09-16

**Authors:** David Evans, Jessica Zering, Erica Stohs, Marisa D’Angeli, Kamenar Katarina

**Affiliations:** Emory University, Rollins School of Public Health; University of Nebraska Medical Center; Washington State Department of Health

## Abstract

**Background:** An overall decrease of prescriptions for outpatient fluoroquinolones (FQs) following the FDA’s release of boxed warnings in 2016 was shown in a previous analysis1. An additional study found that this decline held true when further broken down by specialty 2. We sought to determine which different clinician specialties in Washington State (WA) continue to prescribe FQs and if these rates are congruent with previously conducted national analyses. **Methods:** We conducted a comprehensive analysis of Medicare Part D data from 2021. We identified specialty types contributing the highest proportions of FQ claims from the total volume of claims in 2021. Subsequently, we calculated the average FQ claims per 1,000 Medicare Part D beneficiaries for each specialty. The analysis excluded providers with missing beneficiary data. All dosage formulations were included (i.e. topical & oral). We repeated this process for each year from 2021 to 2013 to assess changes in the average FQ prescription rate for each specialty over time. **Results:** Our analysis encompassed 99,250 FQ prescriptions involving 976,209 Medicare Part D beneficiaries from January 1 to December 30, 2021. Among the specialties, urologists emerged as the highest prescribers of FQs to Medicare Part D beneficiaries, closely followed by ophthalmologists and family practitioners (Table 1). Notably, 72.4% of urologists prescribed FQs, while only 12.4% of family practitioners did so in 2021. Trend analysis indicated that the average FQ claims per 1,000 beneficiaries for urologists decreased from 251 claims per 1,000 beneficiaries (SD = 177.98) in 2013 to 130 claims per 1,000 beneficiaries (SD = 122.50) in 2021 (Figure 1A). In contrast, the only specialty types that had positive trends in average FQ claims per 1,000 beneficiaries from 2013 to 2021 included ophthalmologists, optometrists, and otolaryngologists (Figure 1A). **Conclusion:** Throughout the years leading up to 2021, most prescriber specialties contributing high volumes of FQ claims experienced a decline in FQ prescriptions. The positive trend noted amongst ophthalmologists, optometrists, and otolaryngologists could be due to the limited ability to differentiate between oral & topical FQ formulations within the dataset. These findings underscore the importance of understanding specialist prescribing behaviors and partnering with them to formulate tailored antibiotic stewardship guidance. By doing so, we can further promote patient safety and well-being in the context of FQ usage. Public health departments can promote more holistic antibiotic stewardship interventions and better patient safety outcomes with FQs. Ambulatory Fluoroquinolone Use in the United States 2015–2019 Outpatient Fluoroquinolone Prescription Fills.

**Disclosure:** Erica Stohs: Contracted research - Merck; Contracted research – bioMerieux

**Figure:**
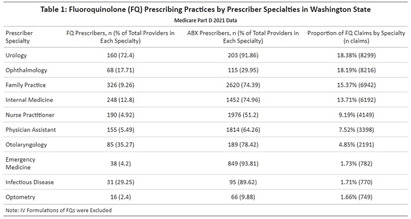

**Figure:**